# Clinical association between trace elements of tear and dry eye metrics

**DOI:** 10.1038/s41598-022-22550-0

**Published:** 2022-10-27

**Authors:** Ying-Jen Chen, Yuan-Yuei Chen, Ching-Huang Lai

**Affiliations:** 1 Department of Ophthalmology, Tri-Service General Hospital; and School of Medicine, National Defense Medical Center, Taipei, Taiwan, Republic of China; 2 Department of Pathology, Tri-Service General Hospital; and School of Medicine, National Defense Medical Center, Taipei, Taiwan, Republic of China; 3 Department of Pathology, Tri-Service General Hospital Songshan Branch; and School of Medicine, National Defense Medical Center, Taipei, Taiwan, Republic of China; 4 School of Public Health, National Defense Medical Center, Taipei, Republic of China

**Keywords:** Environmental sciences, Medical research, Signs and symptoms

## Abstract

Trace elements exposure is proposed to play a role in the pathogenesis of the systemic disease. Emerging studies have suggested that trace metal exposure may contribute to dry eye disease. Our study primarily aimed to investigate the association between trace metal exposure in tear samples and the presence of dry eye metrics in the shipyard industry. Overall, 84 eligible participants from the shipyard industry were included in this cross-sectional study. The parameters for identifying dry eye symptoms included O.S.D.I., SPEED, N.I.B.U.T., and ocular surface conditions, such as tear meniscus height, eye blinking, and meibomian gland area were performed by S.B.M. sistemi ocular surface analyzer. The concentration of tear trace elements was detected by inductively coupled plasma mass spectroscopy (ICP-MS). The association between tear trace elements and dry eye parameters was investigated using regression models. Participants in the exposure group had significantly higher levels of tear Pb than the control group. In the exposure group, tear Pb was significantly associated with increased SPEED and O.S.D.I. score with beta coefficients of 0.144 (95% CI 0.092, 0.197), 0.121 (95% CI 0.049, 0.194), respectively, and decreased lower and upper meibomian gland area with beta coefficients of − 0.158 (− 0.283, − 0.033) and − 0.228 (− 0.396, − 0.061), respectively. Tear trace elements exposure is considered to impact the appearance of dry eye metrics. Improving the occupational environment and monitoring the ocular surface health may benefit workers under exposure to trace elements.

## Introduction

Dry eye disease is one of the most frequent ocular disorders featured with dryness and discomfort^1^. It is a complex and multifactorial disease associated with excessive environmental and biological stress and is a growing public health concern in the world^2,3^. Numerous pieces of evidence have reported the impact of dry eye disease, including aging, gender, lifestyle, antihistamines, and contact lens use^4–6^. The two main categories of dry eye disease are aqueous-deficient and evaporative dry eye^7^. Evaporative type is associated with altered conditions of eyelids, especially meibomian gland dysfunction^8^. Aqueous-deficient type is primarily caused by lacrimal gland dysfunction, such as obstruction and Sjögren's syndrome^9^. Epidemiological studies suggest that the evaporative type is the leading cause of the onset of dry eye disease^10^. However, several underlying pathophysiologies have not yet been explored due to the various potential pathogenic mechanisms involved in developing dry eye disease.

Trace elements are distributed in the environment through natural and artificial processes such as volcanic eruptions, spring waters, air pollution, and industrial processes^11^. They can accumulate in organisms as they are difficult to metabolize, then bind to vital cellular components such as structural proteins, enzymes, and nucleic acids and interfere with their functioning^12,13^. Excessive trace element accumulation is a risk factor for multiple organ damage, even at low levels of exposure^14^.

Emerging evidence has reported the impacts of air pollution, such as particulate matter (PM_2.5_) and trace elements, on the appearance of dry eye disease^15–17^. Increased PM_2.5_ accumulation in the tear film induces ocular surface damage and causes dry eye disease^18,19^. However, information about trace elements analyzed in tear samples is extremely limited due to the difficulty of sampling and low concentration for detection. Welding workers are at risk of trace elements exposure because of the higher frequency of exposure to metal fume in occupational environments^20–22^. In this cross-sectional study, we attempted to investigate the relationships between trace elements in tear samples and dry eye metrics among welding workers from the shipyard industry.

## Method

### Study population

**Figure 1:**
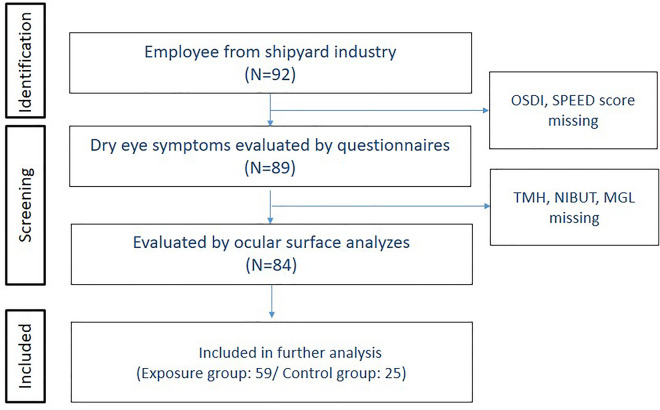
Flow chart of the study.

A longitudinal study has been conducted to explore trace elements' effects on workers in a shipyard since 2014. In 2020, a total of 92 eligible participants aged 20 years or older from a shipyard industry were enrolled for health risk assessment of trace elements of tears during their annual health checkup. (Fig. 1). Comprehensive examinations included self-reported questionnaires, laboratory data, biomarkers, trace elements of tears, and dry eye parameters. Ethics approval was approved by the Institutional Review Board of the Tri-Service General Hospital, Taiwan, and participants provided informed consent before enrollment. Participants who didn't complete the questionnaires for dry eye disease and received ocular surface examination (n = 6) were excluded. The rest of the participants were divided into exposure (n = 59) and control (n = 25) groups based on their work situation^23,24^. The prevalence of dry eye disease was about 1.5% to 4.8% reported by National Health Insurance Research Database in Taiwan^25^. After computing the sample size using a calculator, 73 participants were the minimum number of necessary samples to meet the desired statistical constraints.

### Dry eye parameters

#### Ocular surface disease index (O.S.D.I.)

The precise classification and diagnosis of dry eye disease are complicated by its multifactorial and heterogeneous nature and the variability of symptoms^26^. Consequently, many diagnostic assessments have been proposed to present the condition of the surface layers of the eye^27^. In our study, all procedures were completed by a trained physician. The O.S.D.I. score is a useful and reliable questionnaire providing a rapid evaluation of ocular surface conditions related to dry eye disease^28^. The O.S.D.I. is assessed on a scale of 0 to 100, with higher scores representing greater severity.

#### Standard patient evaluation of eye dryness (SPEED)

Application of the SPEED questionnaire quickly identifies and rates dry eye symptoms by asking four simple questions^29^. A score assesses the SPEED from 0 to 28, which evaluates the severity of dry eye presentations, including dryness, scratchiness, irritation, burning, soreness, and eye fatigue.

#### Ocular surface examination

These examinations were performed by an ocular surface analyzer (S.B.M. Sistemi Ocular Surface Analyzer), a valid and noninvasive device for monitoring ocular surface condition^30^.

#### Noninvasive tear film break-up time (N.I.B.U.T.)

N.I.B.U.T. is a practical and noninvasive method for assessing tear film stability and displays better diagnostic ability than standard TBUT^31^. Many researchers consider N.I.B.U.T. of less than 10 s as dry eye disease^32^. The tear meniscus is a reservoir of tear fluid that contains 75% of the tear volume^33^.

#### Tear meniscus height (T.M.H.)

Evaluation of T.M.H. has been proposed as informative for assessing dry eye disease with relatively high specificity and sensitivity^34^. A normal T.M.H. is between 0.2 and 0.3 mm, and dry eye disease is suggestive of T.M.H. < 0.25 mm^35^.

### Meibography imaging

Quantitative evaluation of meibomian glands is essential in assessing the severity of meibomian gland dysfunction^36^. An emerging study has proposed that images of the meibomian gland are a valuable tool to approach dry eye disease^37^. The upper and lower meibomian gland area ratio to the total area was recorded, respectively.

### Measurement of trace elements in the tear sample

The procedure for collecting tear samples was performed by a capillary tube. We used plastic tubes rather than glass material to minimize the risk of injury. Sampling with capillary tubes collected 10 µl of tear sample. The samples were extracted from the capillaries by centrifugation. The tear sample was collected by capillary action. The trace elements included vanadium (V), chromium (Cr), manganese (Mn), iron (Fe), cobalt (Co), nickel (Ni), copper (Cu), zinc (Zn), arsenic (As), selenium (Se), rhodium (Rh), cadmium (Cd), mercury (Hg), and lead (Pb). We measured the trace elements by Inductively Coupled Plasma Mass Spectroscopy (ICP-MS) (Thermo Fisher iCAP RQ ICA-MS, U.S.A.) based on a previous method^38^. These samples were mixed with concentrated nitric acid (Fisher Scientific, U.K.). Deionized water blanks were used to detect any contamination in the analytical process. A certified rock standard (BCR1) solution was used to check the accuracy of the procedure.

### Measurement of covariates

Basic information, such as age, educational level, and smoking history, was obtained from self-reported questionnaires. Laboratory data, including complete blood count, liver function test, renal function test, and serum lipid profiles, were analyzed by standard protocols. The waist circumference assessment was recorded in the horizontal plane between the iliac crest. The lowest rib and hip circumference was measured around the most comprehensive portion of the buttock.

### Statistical analysis

In terms of statistical analysis, we used the Statistical Package for the Social Sciences, version 22.0 (S.P.S.S. Inc., Chicago, IL, U.S.A.). Pairwise correlation for tear trace elements and dry eye parameter is presented by heatmap illustration. In these trace elements to each dry eye parameter, weighted quantile sum (W.Q.S.) regression was analyzed to assess the highest contributor. The threshold for statistical significance was defined as a *p*-value lower than 0.05. The associations between trace elements in the tear sample and different dry eye parameters were analyzed using a linear regression model, which was adjusted by age, white blood count, creatinine, alanine aminotransferase, and smoking history.

### Ethics approval and consent to participate

We obtained patient permission before enrollment by asking them to complete a written informed consent, and approval for the study was granted by the I.R.B. of Tri-Service General Hospital, Taiwan. In addition, the study design was confirmed in accordance with the Helsinki Declaration.

## Results

### Study population and trace elements

The participants' demographics in each exposure and control group are shown in Table 1. Participants in the exposure group had significantly higher levels of WBC and albumin than the control group. Regarding diagnostic tests for dry eye disease, the Schirmer test in exposure and control was 10.28 ± 7.48 and 9.76 ± 9.07 mm, respectively. In addition, N.I.B.U.T. was significantly higher in the exposure group than in the control group (*p* = 0.005). However, other dry eye parameters such as SPEED, O.S.D.I. score, meibomian gland area, T.M.H., and Schirmer test had no significant difference between exposure and control groups.

**Table 1:** Characteristics of study population.

Variables	Exposure (N = 59)	Control (N = 25)	*P*-value
**Continuous variables, mean (SD)**			
Age (years)	41.15 (11.76)	51.64 (12.16)	< 0.001
Body mass index (kg/m^2^)	26.13 (3.77)	26.89 (4.32)	0.426
Systolic blood pressure (mmHg)	141.83 (23.90)	141.64 (24.47)	0.974
Diastolic blood pressure (mmHg)	80.83 (15.84)	79.48 (14.28)	0.714
Heart rate (bpm)	87.14 (14.73)	85.35 (17.10)	0.662
Waist circumference (cm)	91.14 (8.71)	92.74 (12.34)	0.502
Hip circumference (cm)	102.32 (7.65)	102.08 (8.48)	0.898
Hemoglobin (g/dL)	15.23 (1.13)	14.94 (1.40)	0.321
Platlet (10^3^/uL)	274.88 (67.42)	262.20 (63.66)	0.425
BUN (mg/dL)	14.46 (3.19)	14.76 (4.07)	0.720
Creatinine (mg/dL)	0.93 (0.13)	1.00 (0.39)	0.243
Cholesterol (mg/dL)	202.05 (56.69)	181.84 (26.54)	0.093
Triglycerides (mg/dL)	159.14 (198.93)	106.08 (51.79)	0.194
HbA1c (%)	5.61 (0.91)	5.49 (0.42)	0.523
Uric acid (mg/dL)	6.24 (1.23)	6.14 (1.31)	0.740
Amylase (mg/dL)	70.53 (22.17)	74.16 (29.19)	0.535
AST (U/L)	25.80 (7.39)	27.40 (11.24)	0.442
ALT (U/L)	32.22 (16.56)	31.76 (18.87)	0.911
Alkaline Phosphatase (U/L)	70.22 (16.44)	70.00 (18.93)	0.957
Total bilirubin (mg/dL)	0.91 (0.45)	0.93 (0.31)	0.816
Direct bilirubin (mg/dL)	0.31 (0.15)	0.32 (0.13)	0.612
γ-GT (U/L)	39.53 (37.26)	32.16 (21.65)	0.359
Lactic dehydrogenase (U/L)	177.51 (28.75)	184.88 (36.48)	0.325
**Dry eye test**			
SPEED score	4.76 (4.90)	4.72 (3.53)	0.969
OSDI score	6.98 (5.85)	7.72 (5.95)	0.601
NIBUT	7.18 (0.95)	6.46 (0.99)	0.005
Meibomian gland area	14.61 (8.93)	18.28 (12.80)	0.136
TMH (mm)	0.17 (0.10)	0.16 (0.08)	0.276
Schirmer test (mm)	10.28 (7.48)	9.76 (9.07)	0.606
**Category variables, (%)**			
Education (> high school)	53 (91.4)	25 (100)	0.749
Smoking history	29 (49.1)	13 (52.0)	0.799
Secondary smoking	30 (50.8)	7 (28.0)	0.055

SPEED, standard patient evaluation of dry eye; OSDI, ocular surface disease index; NIBUT, noninvasive tear break up time; MGL, meibomian gland loss; TMH, tear meniscus height.

The concentration of trace elements in the tear sample for each exposure and control group is presented in Table 2. Participants in the exposure group had significantly higher levels of Pb (*p* = 0.016) than the control group.

**Table 2:** Characteristics of tear trace elements.

Variables	Exposure (N = 59)	Control (N = 25)	*P*-value
**Tear trace elements, mean (SD)**			
V (ng/mL)	1.04 (1.37)	0.79 (0.76)	0.301
Cr (ng/mL)	33.68 (42.52)	25.09 (22.73)	0.232
Mn (ng/mL)	2.69 (2.01)	3.23 (2.98)	0.406
Fe (ng/mL)	349.61 (558.71)	298.59 (332.52)	0.605
Co (ng/mL)	0.68 (0.42)	0.62 (0.32)	0.420
Ni (ng/mL)	183.65 (345.88)	112.21 (198.51)	0.236
Cu (ng/mL)	52.28 (72.26)	60.65 (163.01)	0.806
Zn (ng/mL)	303.07 (787.94)	187.54 (447.67)	0.397
As (ng/mL)	0.16 (0.17)	0.13 (0.08)	0.286
Se (ng/mL)	9.64 (2.37)	8.83 (0.84)	0.123
Rh (ng/mL)	418.65 (109.75)	379.15 (112.62)	0.143
Cd (ng/mL)	1.40 (1.29)	1.04 (0.73)	0.117
Hg (ng/mL)	1.42 (2.05)	1.12 (1.71)	0.491
Pb (ng/mL)	23.58 (55.15)	3.42 (20.33)	0.016

SD, standard deviation; V, vanadium; Cr, chromium; Mn, manganese; Fe, iron; Co, cobalt; Ni, nickel; Cu, copper; Zn, zinc; As, arsenic; Se, selenium; Rh, rhodium; Cd, cadmium; Hg, mercury; Pb, lead.

### Correlation of tear trace elements and dry eye parameters

**Figure 2:**
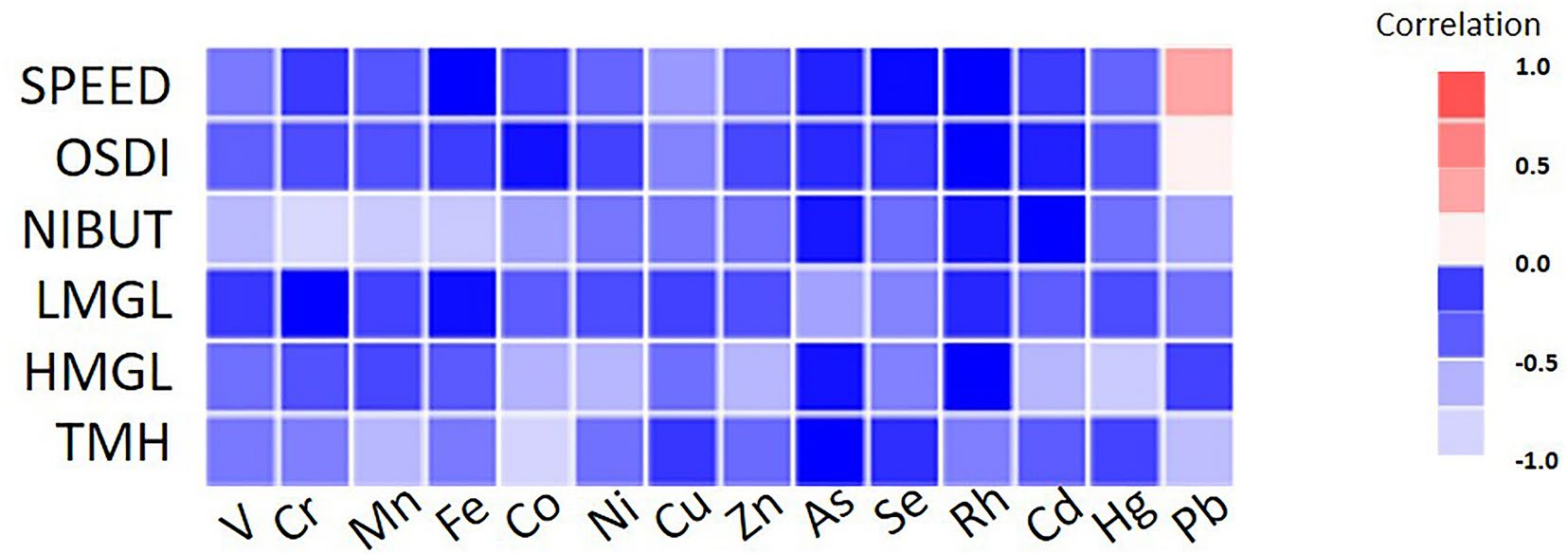
Heatmap illustration presents a pairwise correlation between tear trace elements and dry eye disease parameters.

A Heatmap illustration of pairwise correlation for tear trace elements and dry eye parameters is shown in Fig. 2. The concentrations of Pb in tears had a moderate correlation with the SPEED score and a mild correlation with the O.S.D.I. score. The correlations between different trace elements and dry eye parameters were generally weak.

### Association between Pb and dry eye parameters

**Figure 3:**
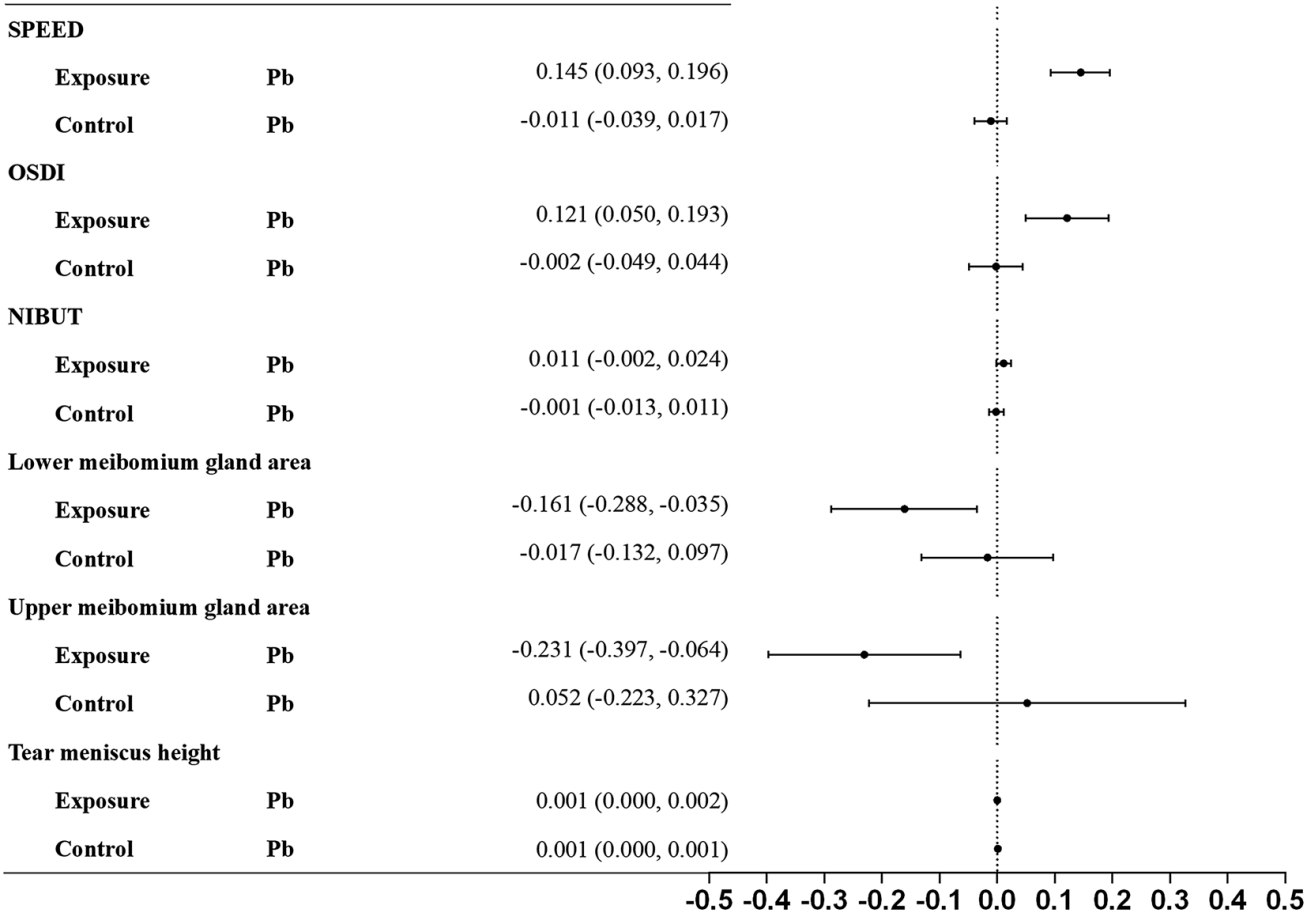
Associations between tear Pb and dry eye disease parameters in each exposure and control group.

The relationships between tear Pb and dry eye parameters were demonstrated in Fig. 3. In the fully adjusted model, Pb was significantly associated with increased SPEED. O.S.D.I. score with beta coefficients of 0.145 (95% CI 0.093, 0.196), 0.121 (95% CI 0.050, 0.193), respectively, and decreased lower and upper meibomian gland area with beta coefficients of − 0.161 (95% CI − 0.288, − 0.035) and − 0.231 (95% CI − 0.397, − 0.064), respectively in exposure group. Nevertheless, the association between tear Pb and dry eye parameters in the control group fails to achieve a statistically significant difference.

### The contributions of tear Pb in dry eye parameters

**Figure 4:**
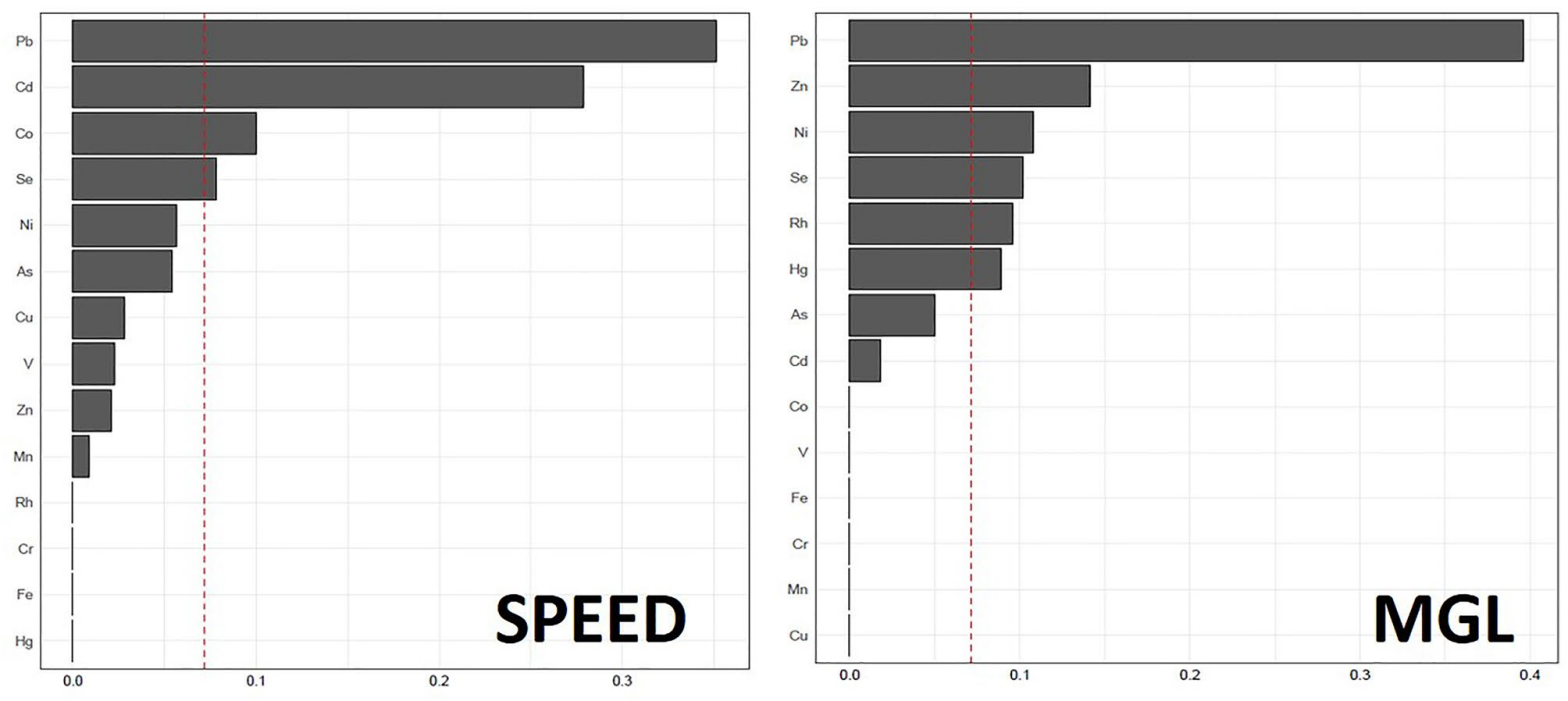
Associations between tear trace elements and dry eye disease parameters based on weighted quantile sum analysis.

Figure 4 shows the contribution of tear Pb to each dry eye parameter individually by weighted quantile sum regression model. Pb is the highest contributor to SPEED and meibomian gland area. Significant associations between the trace elements and the dry eye parameters are noted based on the W.Q.S. regression models.

## Discussion

Our study elucidated the relationship between tear trace elements and dry eye parameters. Tear Pb was significantly associated with increased SPEED, O.S.D.I. score, and decreased meibomian gland area. In addition, Chung et al. reported that mercury concentration in blood was significantly associated with dry eye diseases^39^. Our research survey was the first to use trace elements concentration in tear samples to analyze their effect on dry eye parameters.

Trace elements exposure has been proven to affect many kinds of ocular diseases. Toxic trace elements such as Pb, Hg, and Cd were demonstrated to harm age-related macular degeneration in a Korean population^40^. Vennam et al. reported the neurotoxicity for trace elements such as As, Cd, and Pb that contribute to glaucoma^41^. Pb and Cd have been found in human ocular tissue, especially in the retinal pigment epithelium and choroid^42^. Accumulating these trace elements might damage the neuroprotective functions of the retina and lead to age-related macular disease^43^. Recent studies have indicated that inflammation in the lacrimal glands and ocular surface may contribute to dry eye disease via decreased aqueous tear secretion, conjunctival goblet cell apoptosis, and meibomian gland disruption^44–46^. We proposed that Pb, especially in the tear sample, was substantially correlated with dry eye parameters.

The impaired function of the meibomian gland represents one of the leading conditions that cause dry eye disease^47^. Disruption of meibomian gland function impacts both the quality and quantity of meibum secretion, affecting ocular surface health through changes in tear film composition^48^. The current studies have demonstrated that air pollution, especially PM_2.5_, may promote the development of dry eye disease and meibomian gland dysfunction by regulating a series of inflammatory process^49^. The production of reactive oxygen species from these trace elements is essential in developing eye diseases^50,51^. Our research found that tear Pb was significantly correlated with meibomian gland dysfunction. Therefore, we speculated that trace elements might influence the ocular surface condition by the same pathway of air pollution. Some plausible mechanisms were proposed for the exposure of Pb to dry eye parameters. First, Pb exposure is associated with adverse changes in inflammatory markers leading to accelerated inflammatory response^52^. Next, hyperosmolarity of tear film might explain the relationship of Pb with dry eye disease. Hyperosmolarity of the tear film is regarded as one of the primary mechanisms involving dry eye disease^53^. Last, oxidative stress is suggested to be associated with increased osmolarity of tear film^54^. The free radical reaction generated by Pb exposure might cause hyperosmolarity, then lead to the manifestations of dry eye disease.

Several limitations should be concerned in this study. First, a causal effect relationship cannot be interpreted due to the cross-sectional design. A population-based longitudinal study is needed for further evaluation. Second, the analysis did not adjust some risk factors to confound the relationship between dry eye disease and tear Pb, including using contact lenses or systemic drugs. Next, this study did not record the period for individuals who had exposure to trace elements in the working area. Last, another limitation of this study was the small number of cohort samples. Further studies, including more tear samples from participants' exposure to trace metal, need to apply these markers in diagnosis.

## Conclusion

The present study highlighted the significant relationship between occupational exposure to Pb with dry eye metrics. Further experimental and longitudinal research was warranted to elucidate a causal association between trace elements exposure and dry eye metrics. Our study also suggests that public health intervention is needed to control occupational pollution.

## Supplementary Information

Supplementary Information.

## Data Availability

The datasets during the current study are not publicly available due to the consent requirement of participants, but sex and age decade-stratified descriptive data are available from the corresponding author upon reasonable request.

## Abbreviations

O.S.D.I.: Ocular surface disease index
SPEED: Standard patient evaluation of eye dryness
N.I.B.U.T.: Noninvasive tear film break-up time
T.M.H.: Tear meniscus height
V: Vanadium
Cr: Chromium
Mn: Manganese
Fe: Iron
Co: Cobalt
Ni: Nickel
Cu: Copper
Zn: Zinc
As: Arsenic
Se: Selenium
Rh: Rhodium
Cd: Cadmium
Hg: Mercury
Pb: Lead
