# Supplementary material for: Clinical association between trace elements of tear and dry eye metrics
Source: Sci Rep. 2022 Oct 27;12:18052. doi: 10.1038/s41598-022-22550-0 (PMC9613709; doi:10.1038/s41598-022-22550-0)
Supplement: Supplementary file 1 — Supplementary Information. [file 41598_2022_22550_MOESM1_ESM.docx]

**Supplementary Table 1. Association between variables and dry eye disease parameters**

| **Variables** | **Exposure** | **Control** |
| --- | --- | --- |
|  | **SPEED** | |
| **Age** | -0.046 (-0.136, 0.044) | 0.149 (0.028, 0.270) |
| **WBC** | 0.156 (-0.592, 0.904) | -0.645 (-1.608, 0.317) |
| **Cr** | 4.445 (-3.623, 12.513) | 3.738 (-0.125, 7.601) |
| **ALT** | -0.011 (-0.075, 0.052) | 0.075 (-0.012, 0.162) |
| **Smoking** | 0.979 (-0.256, 2.214) | 2.051 (0.246, 3.857) |
| **Pb** | 0.144 (0.092, 0.197) | -0.014 (-0.042, 0.013) |
|  | **OSDI** | |
| **Age** | -0.070 (-0.195, 0.055) | 0.231 (0.040, 0.422) |
| **WBC** | -0.006 (-1.043, 1.030) | -1.514 (-3.039 |
| **Cr** | 5.960 (-5.218, 17.138) | 5.704 (-0.414, 11.821) |
| **ALT** | -0.026 (-0.114, 0.062) | 0.076 (-0.061, 0.213) |
| **Smoking** | 1.285 (-0.426, 2.997) | 3.765 (0.905, 6.624) |
| **Pb** | 0.121 (0.049, 0.194) | -0.010 (-0.053, 0.034) |
|  | **NIBUT** | |
| **Age** | -0.013 (-0.036, 0.010) | -0.012 (-0.063, 0.039) |
| **WBC** | -0.124 (-0.315, 0.066) | -0.054 (-0.412, 0.305) |
| **Cr** | 1.702 (-0.305, 3.709) | 0.325 (-1.201, 1.852) |
| **ALT** | -0.006 (-0.022, 0.009) | -0.003 (-0.038, 0.032) |
| **Smoking** | 0.019 (-0.291, 0.329) | 0.217 (-0.554, 0.988) |
| **Pb** | 0.011 (-0.001, 0.024) | -0.002 (-0.014, 0.011) |
|  | **Lower meibomium gland** | |
| **Age** | 0.055 (-0.162, 0.271) | 0.295 (-0.230, 0.820) |
| **WBC** | -1.390 (-3.184, 0.404) | 0.308 (-3.880, 4.497) |
| **Cr** | 11.202 (-8.146, 30.549) | 5.552 (-11.259, 22.364) |
| **ALT** | 0.097 (-0.055, 0.249) | -0.004 (-0.380, 0.373) |
| **Smoking** | 1.996 (-0.967, 4.958) | 5.307 (-2.551, 13.165) |
| **Pb** | -0.158 (-0.283, 0.033) | -0.016 (-0.135, 0.104) |
|  | **Upper meibomium gland** | |
| **Age** | 0.167 (-0.123, 0.457) | 0.094 (-1.075, 1.262) |
| **WBC** | -1.022 (-3.422, 1.378) | -0.133 (-8.671, 8.405) |
| **Cr** | 4.556 (-21.327, 30.439) | -1.955 (-37.566, 33.656) |
| **ALT** | 0.070 (-0.134, 0.273) | -0.032 (-0.818, 0.755) |
| **Smoking** | 3.427 (-0.536, 7.390) | 1.319 (-15.797, 18.434) |
| **Pb** | -0.228 (-0.396, -0.061) | 0.051 (-0.240, 0.342) |
|  | **Tear meniscus height** | |
| **Age** | -0.001 (-0.003, 0.001) | 0.001 (-0.003, 0.003) |
| **WBC** | 0.001 (-0.013, 0.015) | -0.004 (-0.030, 0.022) |
| **Cr** | -0.061 (-0.212, 0.090) | -0.043 (-0.147, 0.061) |
| **ALT** | 0.001 (-0.001, 0.001) | 0.001 (-0.001, 0.003) |
| **Smoking** | -0.002 (-0.025, 0.021) | -0.007 (-0.055, 0.042) |
| **Pb** | 0.001 (0.000, 0.002) | 0.001 (0.000, 0.001) |
